# The evolutionary conserved FOXJ1 target gene *Fam183b* is essential for motile cilia in *Xenopus* but dispensable for ciliary function in mice

**DOI:** 10.1038/s41598-018-33045-2

**Published:** 2018-10-02

**Authors:** Anja Beckers, Tim Ott, Karin Schuster-Gossler, Karsten Boldt, Leonie Alten, Marius Ueffing, Martin Blum, Achim Gossler

**Affiliations:** 1 Institute for Molecular Biology, OE5250, Hannover Medical School, Carl-Neuberg-Str. 1, 30625 Hannover, Germany; 2 Institute of Zoology, University of Hohenheim, Garbenstraße 30, 70593 Stuttgart, Germany; 3 Institute of Ophthalmic Research, Center for Ophthalmology, University of Tübingen, Röntgenweg 11, 72076 Tübingen, Germany

## Abstract

The transcription factor FOXJ1 is essential for the formation of motile cilia throughout the animal kingdom. Target genes therefore likely constitute an important part of the motile cilia program. Here, we report on the analysis of one of these targets, *Fam183b*, in *Xenopus* and mice. *Fam183b* encodes a protein with unknown function which is conserved from the green algae *Chlamydomonas* to humans. *Fam183b* is expressed in tissues harbouring motile cilia in both mouse and frog embryos. FAM183b protein localises to basal bodies of cilia in mIMCD3 cells and of multiciliated cells of the frog larval epidermis. In addition, FAM183b interacts with NUP93, which also localises to basal bodies. During frog embryogenesis, *Fam183b* was dispensable for laterality specification and brain development, but required for ciliogenesis and motility of epidermal multiciliated cells and nephrostomes, i.e. the embryonic kidney. Surprisingly, mice homozygous for a null allele did not display any defects indicative of disrupted motile ciliary function. The lack of a cilia phenotype in mouse and the limited requirements in frog contrast with high sequence conservation and the correlation of gene expression with the presence of motile cilia. This finding may be explained through compensatory mechanisms at sites where no defects were observed in our FAM183b-loss-of-function studies.

## Introduction

Cilia are microtubule-based organelles that protrude from the cell surface. Cilia are categorised as non-motile (also called primary) or motile^1^. Virtually every vertebrate cell carries at some point a primary cilium, which is critical for sensing of external stimuli and signal transduction (reviewed in^2,3^). In contrast, motile cilia are present on specialised cell types; they either move cells through the surrounding medium or extracellular fluids along the cell surface (e.g.^4–6^). Motile cilia are essential for normal embryonic development and for function of a multitude of tissues. Single motile cilia on cells of the left-right organiser (LRO) of vertebrate embryos rotate and thereby generate a leftward fluid flow, which is essential for the asymmetric development of visceral organs^7–9^. Multiple motile cilia that are present on the surface of epithelial cells of the respiratory tract, the fallopian tube, or on ependymal cells lining the ventricles, beat in a coordinated manner. They thereby clear the airways from inhaled particles or pathogens^5,10^, support the movement of eggs into the ampulla of the oviduct and towards the uterus^11^, or contribute to the cerebrospinal fluid flow^6,12–14^. The highly specialised motile cilium of the sperm cell, the flagellum, is essential for sperm motility and fertilisation^4^. Dys- or impaired function of motile cilia in humans lead to a condition referred to as primary cilia dyskinesia (PCD). PCD patients suffer from impaired mucociliary clearance and respiratory problems. Accompanying defects may include organ situs randomisation associated with cardiac malformations, male infertility, and - less frequently - reduced female fertility and hydrocephalus (reviewed in^15^), although the latter is frequently found in mouse models of PCD^14^. Mucociliary epithelia are also encountered outside of the airways. For example, as a first line of defense, multiciliated cells (MCCs) in the *Xenopus* tadpole skin move mucus across the epithelium to keep it free from pathogens^16^.

In vertebrates, the development of motile cilia depends critically on the transcription factor FOXJ1^17–23^. FOXJ1 activates the transcription of numerous known ciliary genes^6,20,21,24–28^ and acts downstream of NOTO in the mouse LRO^18,29^. Genes with unknown function that act downstream of FOXJ1 are likely also required for the formation or function of motile cilia. In microarray screens for FOXJ1 target genes, we have identified *Fam183b* as one such candidate gene^28^.

Here, we describe the expression and functional analysis of *Fam183b* in frog embryos and mice. In both species, expression was correlated with the presence of motile cilia. FAM183b protein localised to the basal body and interacted with nucleoporin NUP93, which also localises to the basal body. Knockdown of the *Fam183b* ortholog in *Xenopus* embryos resulted in severely impaired ciliary movement in the tadpole skin. Surprisingly, a null allele in mice had no obvious cilia-related phenotypes, suggesting the presence of mechanisms that effectively compensate the loss of FAM183b in mice.

## Results

### Expression and subcellular localisation of *Fam183b*

**Figure 1:**
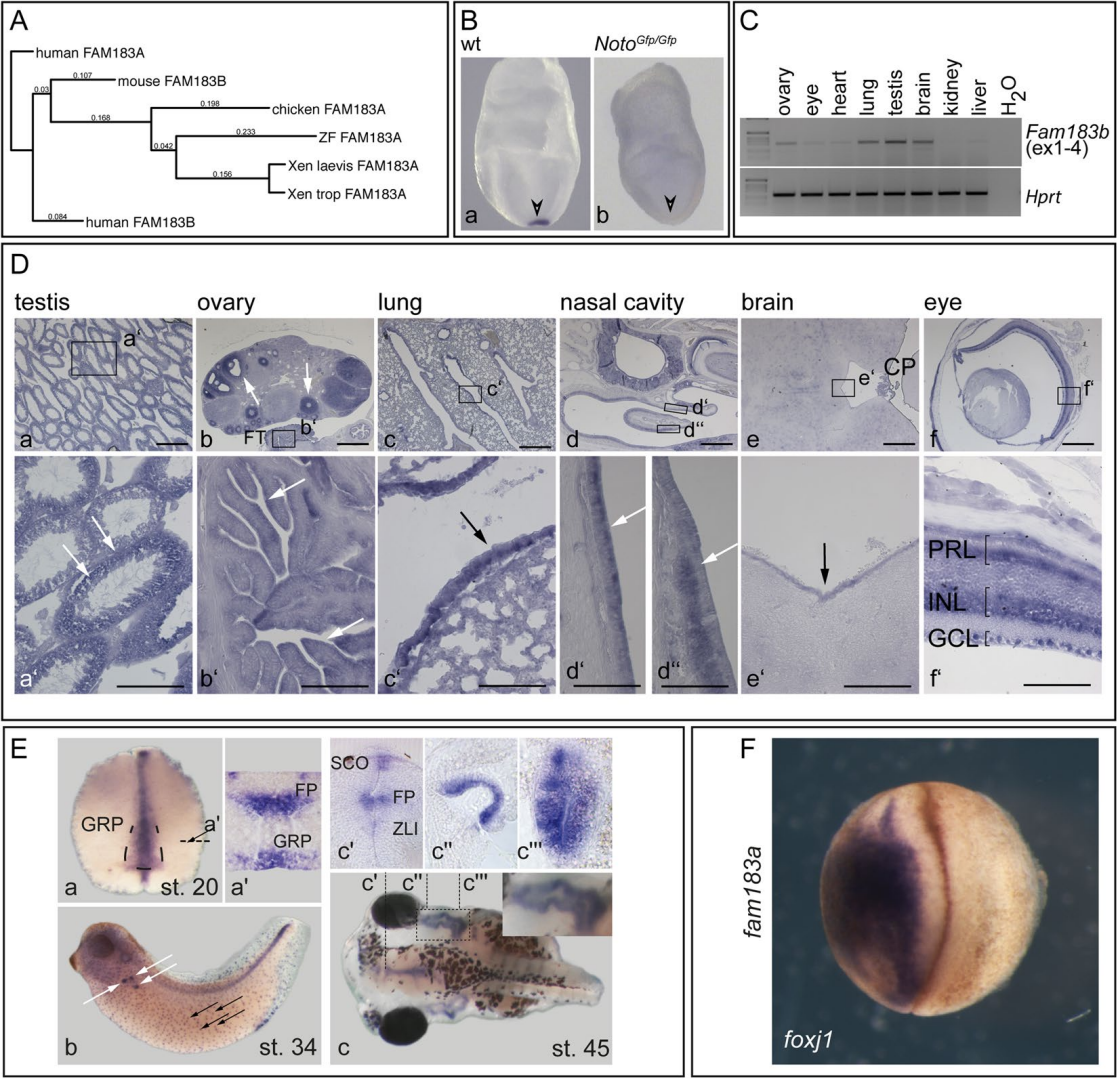
Expression of *Fam183b*. (**A**) Phylogram of vertebrate *Fam183* genes rooted on human *FAM183A*. Sequences used for alignment and phylogenetic analysis: human, HGNC-34347 and HGNC-34511; mouse, Q5NC57; chicken, F1P3Y5; Xen. tropicalis, XP_004914015; Xen. laevis, XP_018113781; zebrafish, ZDB-GENE-111103-1. (**B**) WISH of E7.75 wild type (wt) (a) and *Noto*^*Gfp/Gfp*^ (b) mutant embryos shows NOTO-dependent expression of *Fam183b* in the LRO. (**C**) Analysis of *Fam183b* expression by RT-PCR of RNA from adult organs, as indicated. Full size gel is shown in Supplementary Figure S1. (**D**) SISH analysis of adult tissues, as indicated. Boxed areas in a–f outline regions shown at higher magnification in a’–f’. Arrows point to sites of expression. FT: fallopian tube; CP: choroid plexus; PRL: photoreceptor layer; INL: inner nuclear layer; GCL: ganglion cell layer. (**E**) Expression of *fam183a* in *Xenopus laevis*. *Fam183a* mRNA was found at stage 20 in the floor plate (FP) and LRO (GRP; a,a’); at stage 34 in MCCs and nephrostomes (b); and at stage 45 (c) in the sub-commissural organ (SCO), the zona limitans intrathalamica (ZLI) and the floor plate of the brain (c’), in the dorsal lining of the branchial chamber (inset in c and c”), and the stomach (c”’). (**F**) *Fam183a* is a *foxj1* target gene. Embryos were unilaterally injected on the left side with a *foxj1 mRNA* and analysed for *fam183a* expression. *foxj1*, injected side. Scale bars: Da–f = 500 μm, a’–f’ = 100 μm.

*Fam183b* encodes a protein of 137 amino acids with unknown biochemical function. FAM183b is homologous to the flagellar associated protein FAP144 (ACJ06133.1) of *Chlamydomonas* and highly conserved from cnidarians to mammals (Supplementary Table S1). Most vertebrate genomes harbor one Fam183 gene, which in the mouse is called *Fam183b*, while in all other species it is referred to as *Fam183a*. The human genome is the only case in which two Fam183 genes have been annotated (Fig. 1A), which are referred to as FAM183A (hgnc_id = HGNC:34347) and FAM183B (hgnc_id = HGNC:34511). The latter represents a pseudogene (https://www.genenames.org/cgi-bin/search?search_type=all&search=fam183b&submit=Submit). Thus, the mouse *Fam183b* gene constitutes the ortholog of *Fam183a* genes in other vertebrates and humans (http://www.informatics.jax.org/marker/MGI:1922679).

In early mouse embryos, *Fam183b* expression, detected by whole mount *in situ* hybridisation (WISH), was confined to the LRO at E7.75 (node; arrowhead in Fig. 1Ba). In *Noto-*mutant E7.75 embryos, transcripts were not detected (Fig. 1Bb), indicating that *Fam183b* acts downstream of NOTO. Absence of *Fam183b* transcripts may result from down-regulation of *Foxj1* in *Noto* mutants, as NOTO acts upstream of FOXJ1 in the LRO^18^ and *Fam183b* is down-regulated in the *Foxj1*-mutant foetal respiratory epithelium^28^. In adult mice, strong *Fam183b* expression was detected by RT-PCR in tissues carrying motile cilia, while lower transcript levels were found in a few additional tissues such as eye and heart (Fig. 1C). Strong expression in cells carrying motile cilia was consistently seen upon *in situ* hybridisation of histological tissue sections, i.e. in spermatogenetic cells in the testis (Fig. 1Da, arrows in a’), multiciliated cells of the fallopian tube (arrows in Fig. 1Db’), the respiratory tract (Fig. 1Dc, arrow in c’, d, arrows in d’, d’’) as well as the choroid plexus (CP; Fig. 1De) and ependymal layer of the brain (Fig. 1De, arrow in e’). Notable exceptions from this correlation were found in developing follicles in the ovary (arrows in Fig. 1Db) and in the eye, where photoreceptor cells and cells of the inner nuclear and ganglion cell layers of the retina displayed *Fam183b* mRNA staining (Fig. 1Df,f’). *In situ* hybridisation of *Xenopus laevis* embryos revealed *fam183a* expression at stage 20 in the gastrocoel roof plate (GRP; Fig. 1Ea,a’), i.e. the *Xenopus* LRO^30^ and in the floor plate of the neural tube (FP; Fig. 1Ea’). At stage 34, transcripts were detected in the ciliated nephrostomes (white arrows in Fig. 1Eb) and epidermal MCCs (black arrows in Fig. 1Eb). Stage 45 tadpoles revealed *fam183a* gene expression in the sub-commissural organ, the zona limitans intrathalamica and the floor plate of the brain (SCO, ZLI, and FP; Fig. 1Ec’), in the dorsal epithelium of the branchial chambers (inset in Fig. 1Ec and Fig. 1Ec”), and in the stomach (Fig. 1Ec”’). Overexpression of *foxj1* on the left side of *Xenopus* embryos induced ectopic expression of *fam183a* (Fig. 1F), corroborating the requirement of FOXJ1 for *Fam183b* expression in mice (Fig. 1B and^28^) and further supporting the notion that FAM183b acts downstream of FOXJ1 and NOTO.

**Figure 2:**
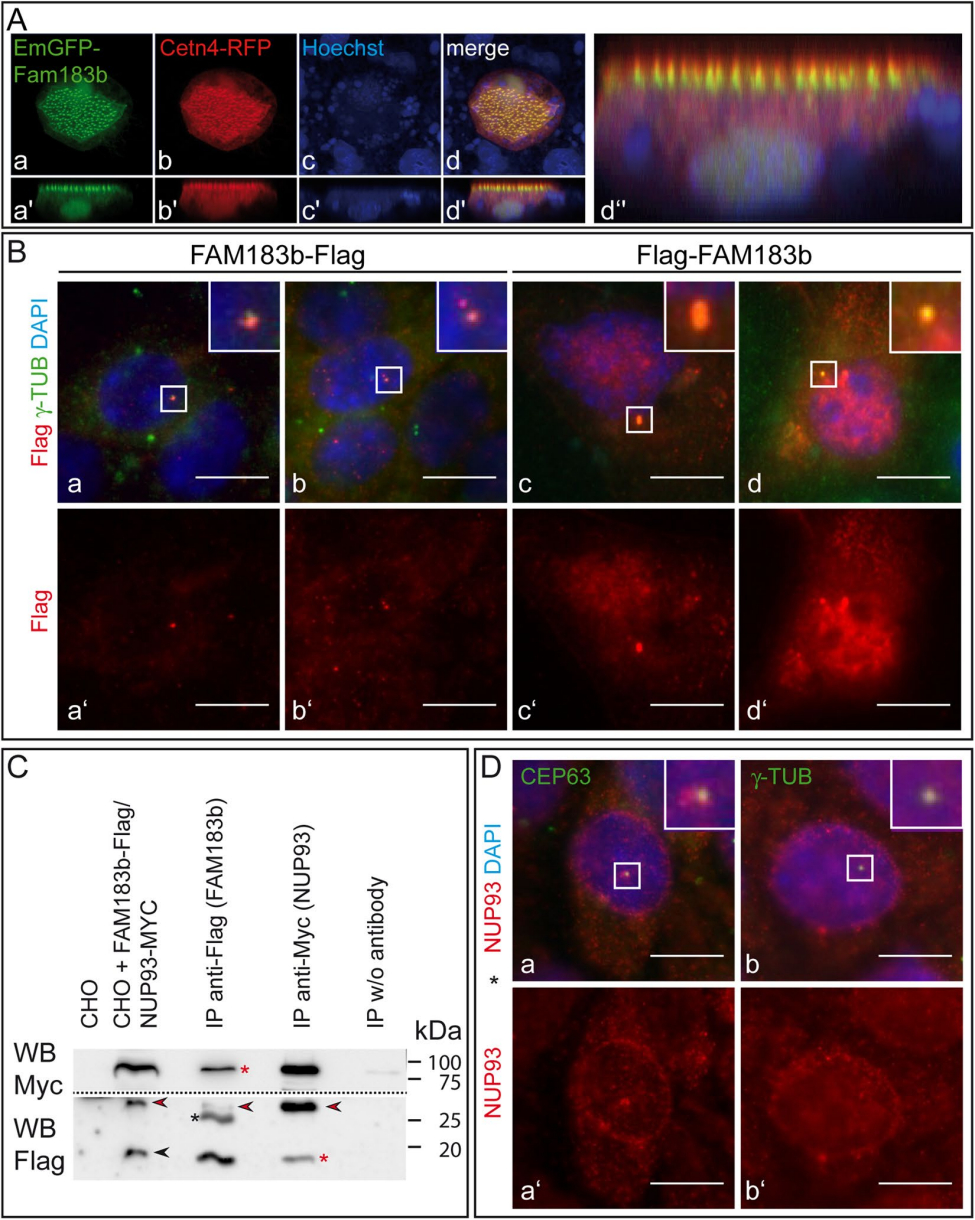
Subcellular localisation of FAM183B. (**A**) Co-localisation of mouse FAM183b-GFP with centrin4 in stage 33 *Xenopus* embryos. (**B**) Detection of C- and N-terminally Flag-tagged FAM183b in mIMCD3 cells by indirect immunofluorescence showing partial overlap with γ-tubulin. (**C**) Co-IP of tagged FAM183b and NUP93, indicating interaction. Red asterisks: co-IP; black asterisk: anti-Flag light chain detected by secondary antibody; black arrowhead: FAM183b at the expected apparent molecular weight; red arrowheads: background band detected in transfected CHO cells and IPs. Full size Western blots are shown in Supplementary Figure S2. (**D**) Co-localisation of endogenous NUP93 with CEP63 and γ-tubulin at centrosomes. Scale bars: Ba–d,a’–d’, Da,b,a’,b’ = 10 μm.

Antibodies generated against a peptide of mouse FAM183b did not detect the endogenous protein by indirect immunofluorescence in cells or on tissue sections (not shown). In order to investigate the subcellular localisation of FAM183b, we expressed N-terminally GFP-tagged mouse FAM183b in *Xenopus* embryos and observed that tagged FAM183b localised to basal bodies, as shown by co-staining with centrin4 (*Xenopus*, Fig. 2A). In contrast, C- and N-terminally Flag-tagged FAM183b expressed in mIMCD3 cells, which were induced to form (primary) cilia, only partially colocalised with the centrosome/basal body marker γ-tubulin (Fig. 2B), and individual mIMCD3 cells with strong overexpression of FAM183b displayed staining throughout the cell (Fig. 2Bc,c’,d,d’). Collectively, these data demonstrate that expression of FAM183b in tissues carrying motile cilia is conserved between mouse and frog, suggesting a conserved ciliary function in these two vertebrate model organisms.

### *Fam183b* interaction partners

In a first attempt to approach the unknown biochemical function of FAM183b, we set out to identify interaction partners. To that end, a yeast-two-hybrid (Y2H) screen was performed using a testis library. In addition, SF-TAP-tagged^31^ FAM183b was expressed in HEK293 cells, affinity purified, and analysed by liquid chromatography fractionation in combination with mass spectrometry (LC-MS). Non-promiscuous candidate interaction partners identified in the Y2H screen were NUP93, TMEM269 and ANKRD36 (Supplementary Table S2). NUP93 was identified by LC-MS as well, in addition to chaperones, mitochondrial, ribosomal and other proteins not related to motile cilia or centrosomes (Supplementary Table S3). Co-immunoprecipitation (IP) assays using CHO cells co-expressing Flag-tagged FAM183b and Myc-tagged NUP93 confirmed the interaction of FAM183b with NUP93 (Fig. 2C). The centrosomal localisation of endogenous NUP93 in mIMCD3 cells further supported this notion (Fig. 2D). Localisation of FAM183b was not observed at the nuclear envelop of mIMCD3 cells, even in cells strongly overexpressing Flag-tagged-FAM183b (Fig. 2Bc,d). These data suggested that interaction of NUP93 with FAM183b was confined to the centrosome.

### Functional analysis of *fam183a* in *Xenopus laevis*

**Figure 3:**
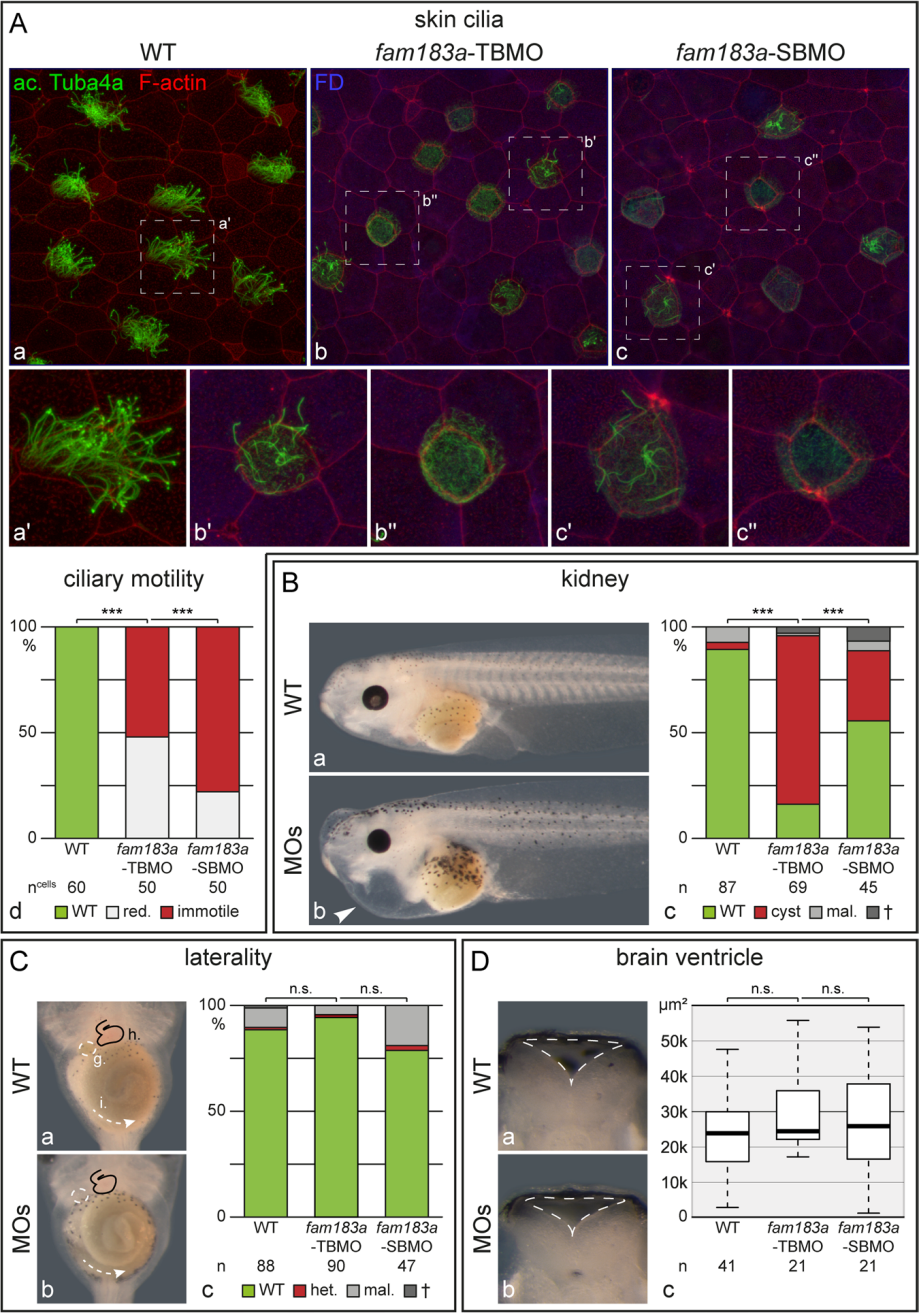
Functional analysis of *fam183a* in *Xenopus laevis*. (**A**) Embryos at the 4-cell stage were injected with *fam183a*-TBMO (b) or -SBMO (c), cultured to stage 33 and analysed for epidermal MCC ciliation (a–c) and ciliary beating (d). Note that cilia were reduced in length and number in MCCs of morphant specimens. Stippled boxes in (a–c) indicate the regions shown enlarged (a’–c”). Edemata (**B**), organ situs (**C**) and hydrocephalus (**D**) were analyzed at stage 45. Note that epidermal cilia defects and cardial edemata, were encountered in a statistically significant proportion of specimens, while rare LR defects and hydrocephalus were not significant. White arrowhead in (**B**b) highlights cardial edema; arrangement of inner organs and gut coiling in (**C**a,b) was illustrated by outlines and arrows, respectively; ventricular margins in (**D**a,b) were depicted by strippled outlines. FD: fluorescin dextran; g: gall bladder; h: heart; het., heterotaxia; i: intestine; mal: malformed; red. reduced; Sa: situs ambiguus; Si: situs inversus; Ss: situs solitus; +: dead.

To assess the physiological function of FAM183a in *Xenopus* embryos, we interfered with protein translation using morpholino oligonucleotides (MO) to block translation (TBMO) or splicing of exon 1 (SBMO), respectively. MOs were unilaterally injected into the epidermal lineage at the 4-cell stage, such that the uninjected side served as an internal control. Treated specimens were cultured to stage 33 and analysed for ciliary motility of the epidermal MCCs. Tadpoles at that stage glide across the agar layer of culture dishes using the motility of skin MCCs^32^. Gliding behaviour was pronounced on the uninjected side, while no motility was detectable when specimens were flipped over to the injected side (not shown), indicating that ciliogenesis or ciliary motility were impaired. To assess cilia and their motility directly, high-speed video microscopy was performed on control and injected sides of morphant tadpoles at stage 33. Compared to wild type specimens (Movie S1), ciliation and ciliary motility were severely impaired (p < 0.0001, respectively) on the MO-injected side, with both MOs yielding comparable results (Movies S2, S3). Ciliation of skin MCCs was impaired, with fewer and shorter cilia on morphant MCCs (Fig. 3A). Additional phenotypes observed in the morphants include frequent pericardial edema (arrow head in Fig. 3Bb, p < 0.0001), which could be due to defective motility of nephrostome cilia or alternatively cardiac or lymphatic dysfunction^33^. In contrast, we did not detect evidence of cardiac/gut looping defects or hydrocephalus (Fig. 3C,D, and data not shown; median values for ventricle profiles: WT = 238269 μm^2^, TBMO = 25909 μm^2^ and SBMO = 24454 μm^2^). This may suggest that cilia in the LRO are functioning normally despite the dramatic ciliary phenotype in the embryonic epidermis. However, given that pericardial edema may complicate ascertainment of the cardiac loop, to be certain that global LR patterning is normal would require additional molecular marker analysis or an examination of cilia in the LRO. In addition, the evolution of hydrocephalus in *Xenopus* due to cilia dysfunction is not well understood, so whether normal ventricle size is indicative of normal cilia function remains unclear.

### Functional analysis of *Fam183b* in the mouse

**Figure 4:**
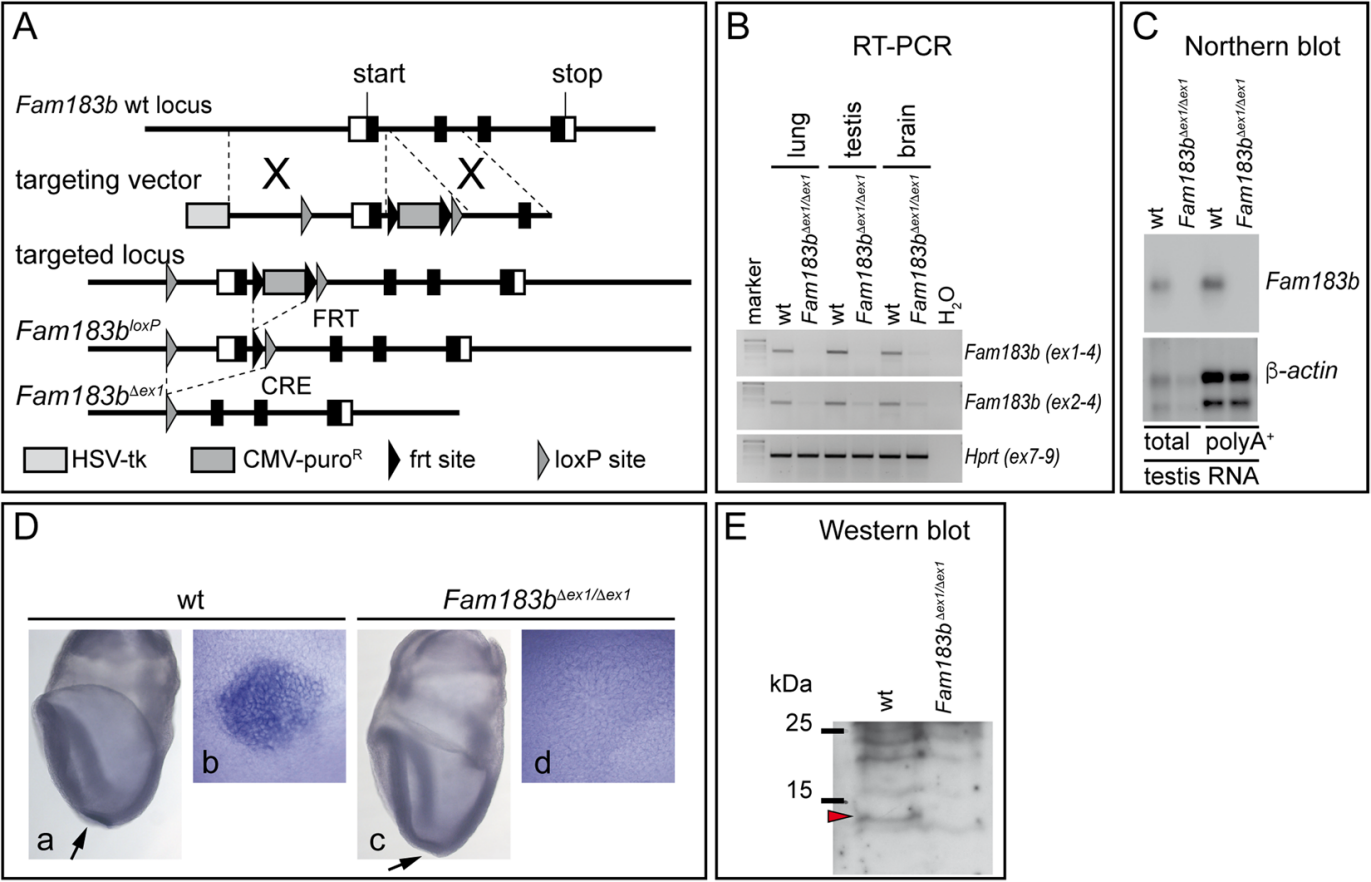
Generation and validation of a *Fam183b*-null allele. (**A**) Schematic drawing depicting the structure of the wild type locus, targeting vector and mutated allele. (**B**) RT-PCR with primers binding in *Fam183b* exon 1 and 4, *Fam183b* exon 2 and 4, and *Hprt* exon 7 and 9 on RNA from adult tissues. Full size gel is shown in Supplementary Figure S3. (**C**) Northern blot of total and polyA^+^ RNA from wt and *Fam183b-*mutant testes. Full size Northern blots are shown in Supplementary Figure S4. (**D**) WISH of E7.75 wt (a,b) and *Fam183b*^*Δex1*/*Δex1*^ (c,d) embryos. (b,d) higher magnification of ventral views of the LRO. (**E**) Western blot analysis of testis lysates from wt and *Fam183b*^*Δex1*/*Δex1*^ testes. The full size Western blot is shown in Supplementary Figure S5.

To analyse the physiological function of FAM183b in mammals, we generated a conditional allele in mice. To that end, two loxP sites were introduced, 1.5 kb upstream of the transcriptional start site into a region of minimal sequence conservation across species, and downstream of exon 1, respectively (Fig. 4A). Cre-mediated recombination deletes the promoter region and transcriptional and translational start sites (*Fam183b*^*Δex1*^) and thus should prevent the translation of FAM183b protein. To generate mice lacking FAM183b in all tissues, we deleted the floxed region of the *Fam183b* locus in the female germ line using *ZP3:Cre* mice^34^. Homozygous *Fam183b*^*Δex1*^ mice were born at Mendelian ratio (7/30) and showed no obvious abnormalities. Loss of *Fam183b* expression was assessed at the RNA level by RT-PCR, Northern blot hybridisation and WISH of early embryos. No transcripts containing exon 1, and only very low amounts of transcripts harbouring exons 2–4 were detected by RT-PCR prepared from various mutant tissues (Fig. 4B). No transcripts were detected in RNA purified from mutant testis by Northern blot hybridisation using a full length *Fam183b* probe (Fig. 4C). *Fam183b* mRNA was not observed by WISH in the LRO of embryos homozygous for the *Fam183b* deletion (Fig. 4D). Our polyclonal anti-FAM183b antibodies, which were directed against a peptide encoded by exon 2, detected a protein of the expected size in the unsoluble fraction of wild type testis lysates (in addition to several background bands), which was missing in lysates from homozygous mutant males (arrowhead in Fig. 4E). Together, these analyses indicated that the *Fam183b*^*Δex1*^ mutation effectively abolished *Fam183b* mRNA transcription and the generation of FAM183b protein, and thus represented a functional null allele.

**Figure 5:**
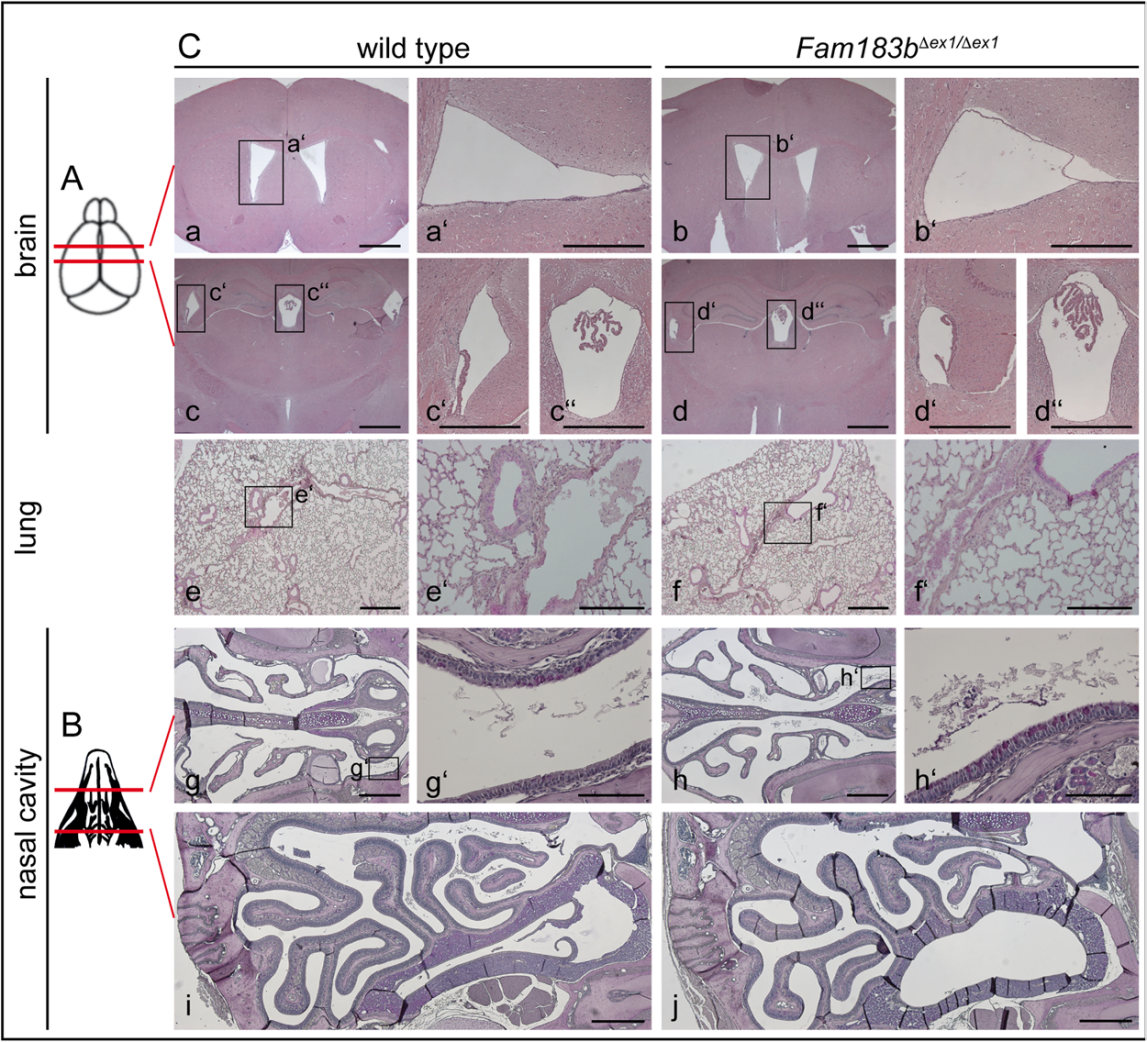
Histological analysis of *Fam183b*-mutant tissues. (**A**) Schematic drawing showing the planes of sections shown in (**C**a–d). (**B**) Scheme depicting planes of sections shown in (**C**g–j). (**C**a–d) Representative sections of wild type (a,c) and *Fam183b*^*Δex1*^ mutant brains (b,d) at the two horizontal levels indicated in (**A**). Boxed areas indicate the regions shown at higher magnification in a’,b’,c’,c’’,d’ and d’’. (**C**e,f) Representative sections of wild type (e) and *Fam183b*^*Δex1*^ mutant (f) lungs. Boxed areas indicate the regions shown at higher magnification in e’,f’. (**C**g–j) Representative sections of wild type (g,i) and *Fam183b*^*Δex1*^ mutant (h,j) nasal cavities at the two horizontal levels indicated in (**B**). Boxed areas indicate the regions shown at higher magnification in g’ and h’. Scale bars: a,b,c,d: 1 mm; a’, b’,c’,c’’,d’,d’’: 500 μm; e,f: 500 μm; e’,f’: 200 μm; g–j: 500 μm; g’,h’: 100 μm.

Male infertility due to immotile spermatozoa, perturbed left-right asymmetry, hydrocephalus and mucus accumulation in the respiratory tract are frequently found in mice with impaired function of motile cilia, whereas female infertility is less common^15^. Homozygous *Fam183b* mutants of both sexes were fertile and showed no externally visible abnormalities over an extended observation period of 12 months (n = 20). Early postnatal lethality that could possibly be caused by cardiac malformations due to situs randomisation was not observed in 10 litters of homozygous breeding pairs. Likewise, inspection of inner organs of 27 homozygous *Fam183b*^*Δex1*^ mice showed no evidence of left-right defects (data not shown). Serially sectioned brains of 6 months old *Fam183b*^*Δex1*^ homozygotes (n = 4) did not reveal enlarged ventricles that might be indicative of hydrocephalus (Fig. 5Cb,b’,d,d’,d’’). Likewise, HE-stained sections of the lung (Fig. 5Cf,f’) or PAS stained sections of the nasal cavities (n = 4) did not show obvious morphological alterations or accumulation of mucus (Fig. 5Ch,h’,j).

Taken together, our functional analyses of *Fam183a/b* in *Xenopus* and mouse revealed striking differences: while the orthologous genes were found to be highly conserved, both in sequence and expression pattern, phenotypic defects were only observed in the frog, where they were restricted to tissues harbouring multiciliated cells.

## Discussion

The ciliation and ciliary motility phenotype encountered upon gene knockdown of *fam183a* in the frog tadpole mucociliary epithelium identifies this novel *Foxj1* target as a potential PCD candidate gene. The lack of a corresponding defect in airway epithelia of knockout mice apparently contradicts this notion. However, this result does not predict in any way whether or not a *Fam183a* null mutation would be causing PCD in human patients. For example, it has been suggested that the phenotypic severity of ciliopathies is a function of the total mutational load in ciliary genes^3^. An inbred mouse strain such as the one used here to create a knockout line in this respect differs greatly from the human population, in which large differences exist with respect to the inter-individual mutational load. It is not unprecedented that mutations in orthologous genes result in different arrays of phenotypes, depending on the model organism in question. For example, null mutations of *Noto* in zebrafish (floating head, *flh*) cause the complete absence of the notochord along the entire anterior-posterior body axis and severe associated patterning defects^35^ whereas *Noto* null-mutant mice show highly variable mild notochord defects restricted to the posterior region^36^. Likewise, various genes that are essential for early steps in neural crest development in non-mammalian vertebrates appear to be dispensable for the corresponding steps in the mouse (reviewed in^37^). Finally, the loss of a functional *Fam183b* allele might be compensated for in the mouse, but not in other vertebrates such as frogs and humans.

Compensation of (germ line) mutations is a phenomenon that is increasingly being observed in various species, the underlying mechanisms being largely unclear (reviewed in^38^). One potential explanation for the lack of an obvious *Fam183b*^*Δex1*^ motile cilia phenotype could be the presence of (a) redundant gene(s) in the mouse. However, we did not find any evidence for the presence of a *Fam183b* paralog in mouse. In zebrafish, *egfl7*-morphants exhibited severe vascular defects while *egfl7*-mutants showed no obvious phenotypes, which was attributed to gene expression changes and consequent compensation in mutant but not knockdown animals^39^. A similar compensatory mechanism might act in mice since siRNA-mediated knock down of *Azi1/Cep131* in embryonic fibroblasts interfered with ciliogenesis, whereas ciliogenesis in *Azi1/Cep131* null-mutant mice as well as null mutant embryonic fibroblasts occurred normally^40^. Given the importance of (motile) cilia for development and maintenance of tissue homeostasis, it seems likely that the function of a number of cilia genes is buffered by compensatory mechanisms to provide genetic robustness. The conditional knockout mouse generated in this study may provide an opportunity to investigate compensatory mechanisms, particularly in the lung.

A direct or indirect interaction of FAM183b with NUP93 is consistent with the localisation of both proteins to basal bodies (Fig. 2B,D) and the reported localisation of NUP93-GFP to the ciliary base^41^. Several nucleoporins including NUP93 have been described to be part of a presumed ciliary pore complex that regulates protein transport into cilia^41^ raising the possibility that FAM183b is involved in this process. Alternatively, nucleoporins may function as scaffolding proteins at the ciliary base, according to a recent study on NUP188 and NUP93, which showed that the nuclear pore was intact in *Xenopus* morphants while biogenesis of motile cilia was impaired^42^. In any case, the identification and verification of NUP93 as a FAM183b interaction partner is in line with a function of this poorly characterised protein in a ciliary context.

It remains to be seen whether human PCD patients with mutations in FAM183A exist or whether humans like mice are able to compensate for the loss of this protein. The high sequence conservation throughout the animal kingdom strongly argues for a function of this gene at least at some of its expression sites. As more examples of transcriptional adaptation to gene loss in the germ line become known, it also becomes increasingly clear that candidate genes for human disease need to be analysed in more than one model organism and using more than one loss-of-function approach. The use of MOs in *Xenopus laevis* may prove to be one such possibility^43^.

## Methods

### Generation and husbandry of animals

Animal experiments were performed in accordance with the German regulations (Tierschutzgesetz). For mice experiments were approved by the ethics committee of Lower Saxony for care and use of laboratory animals (LAVES, Niedersächsisches Landesamt für Verbraucherschutz und Lebensmittelsicherheit), for frogs by the Regional Government Stuttgart, Germany (A379/12 Zo ‘Molekulare Embryologie’). Mice were housed in the animal facility of Hannover Medical School (ZTL), as approved by the responsible Veterinary Officer of the City of Hannover. Animal welfare was supervised and approved by the Institutional Animal Welfare Officer. Frogs were kept at the appropriate condition (pH = 7.7, 20 °C) at a 12 h light cycle in the animal facility of the Institute of Zoology at the University of Hohenheim. Animal welfare was supervised and approved by the Institutional Animal Welfare Officer. Female frogs (4–20 years old) were stimulated with 25–75 units of human chorionic gonadotropin (hCG; Sigma), depending on weight and age, that was injected subcutaneously one week prior to oviposition. On the day prior to ovulation, female frogs were injected with 300–700 units of hCG. Eggs were collected into a petri dish by careful squeezing of the females, followed by *in vitro* fertilization. Sperm of male frogs was obtained by dissecting of testes that were stored at 4 °C in 1x MBSH (Modified Barth’s Saline with HEPES).

*Fam183b*^*loxP*^ mice were generated by Artemis Taconics (Neurather Ring 1, 51063 Köln, Germany). C57BL/6 BAC DNA was used for the generation of the targeting construct. The targeting strategy was based on *Fam183b* NCBI transcript NM_029283_1. Exon 1 (containing the translation initiation codon) and approx. 1.5 kb of sequence upstream of exon 1 (promoter region) was flanked by loxP sites (size of loxP-flanked region: approx. 3.0 kb). The positive selection marker (Puromycin resistance - PuroR) was inserted into intron 1 flanked by FRT sites and was removed by crossing to ACTB::FLPe mice. The phenotype of *Fam183b*^*Δex1*^ mice was analysed on a C57BL/6 (N9) background. Other mouse strains have been described previously: ZP3::Cre^34^, ACTB::FLPe^44^, *Noto*^*Gfp*^ ^29^.

### Genotyping of *Fam183b*^*loxP*^ and *Fam183b*^*Δex1*^ mice

*Fam183b*^*Δex1/*Δ*ex1*^ mice were genotyped by PCR with allele-specific primer pairs:

wild type allele:    Fam183b_35: CAAACAAACCATGTTGCTTGG.

 Fam183b_36: AGTAGAGGCAGGTATGTGTTT  259 bp product.

*Fam183b*^*loxP*^ allele:   Fam183b_35: CAAACAAACCATGTTGCTTGG.

 Fam183b_36: AGTAGAGGCAGGTATGTGTTT  315 bp product.

*Fam183b*^*Δex1*^ allele: Fam183b_35: CAAACAAACCATGTTGCTTGG.

 Fam183b KO 41 R:GAAGGTGAATGGCCTAAATGG 293 bp product.

### Gene knockdown in *Xenopus* embryos

Embryos were injected at the 4 cell stage with *fam183a*-TBMO or *fam183a*-SBMO to target the MCCs (B1/B2 lineage), kidney (C3 lineage), LRO (C1/C2 lineage) and anterior neural tube (A1/A2 lineage). Sequences of MOs were TBMO: 5′-TTAGGCTCGTTTCCAGGGCAACCAT-3′ and SBMO: 5′-TGCCCTCATTCATTACCCTTCCGAT-3′. The following MO-doses were used, which were compatible in all cases with normal development: MCC-injections (TBMO 8 pmol, SBMO 4 pmol); kidney (TBMO 2 pmol, SBMO 1 pmol); LRO (TBMO 2 pmol, SBMO 1 pmol); neural tube (TBMO 2 pmol, SBMO 1 pmol).

### Generation of FAM183b-antibodies and Western blot detection of endogenous FAM183b

A polyclonal anti-FAM183b-antibody was raised in rabbits by immunisation with peptide CTRKPMSWHDNLEEPE (residues 51–65 encoded by exon 2 of mouse FAM183b) and affinity purified by standard procedures. FAM183b-antibodies recognise overexpressed and endogenous protein on Western blots but do not detect FAM183b by immunohistochemistry. For Western blot analyses, one adult testis was lysed in 1 ml RIPA buffer, the non-soluble fraction was sonicated in sample buffer supplemented with 200 mM DTT and 10 mM iodoacetamid and the equivalent of half a testis was loaded into one slot of a 20% PAGE-SDS gel, separated and transferred to Immobilon-P membrane (Merck). Purified antibodies were diluted 1:1000 in 5% milk / 0.5% Tween20 in PBS. Secondary antibodies: anti-rabbit-HRP (GE Healthcare, 1:10000).

### Subcellular localisation of FLAG-tagged FAM183b and endogenous NUP93

N- or C-terminally flag-tagged FAM183b was transiently expressed (Lipofectamine^®^ 2000 Transfection Reagent, Invitrogen) in mIMCD-3 cells (ATCC^®^ CRL-2123^TM^), according to the manufacturer’s instructions. Transfected cells were cultured on gelatinised (0.1% gelatine) cover slips for 16 hrs, fixed with ice-cold methanol and washed three times for 10 min each with PBS. Unspecific antibody binding was blocked for 60 min with 5% FCS in PBS. Cells were incubated with antibodies against flag (mouse anti-flag; Sigma F3165; 1:1000) and γ-tubulin (rabbit anti-γ-tubulin; abcam ab11317; 1:4000). As secondary antibodies, mouse ALEXA-555 (Invitrogen A-31570, 1:200) and rabbit ALEXA-488 (Invitrogen A-11034, 1:200) were used; nuclei were counter-stained with DAPI (0.5 μg/ml, Applichem). Slides were mounted with Prolong Gold Antifade Reagent (Invitrogen) and analysed under a Leica microscope DMI6000 B (100 x oil objective) using LAS AF software. Localisation of mouse NUP93 in mIMCD3 cells was analysed using mouse anti-NUP93 antibody (abcam ab53750; 1:100). Cells were co-stained with rabbit anti-CEP63 (Millipore #06-1292; 1:100) or rabbit anti-γ-tubulin (abcam ab11317; 1:4000). Secondary antibodies used were as described above.

### Subcellular localisation of GFP-tagged FAM183b in *Xenopus*

N-terminally GFP-tagged mouse *Fam183b* (Fam183b in pcDNA6.2/N-EmGFP-Dest; Invitrogen, Gateway system) and cetn4-RFP (cetn4-RFP in pCS2+, to stain basal bodies) plasmids were co-injected into the epidermal lineage of 4-cell frog embryos. Injected specimens were cultured to stage 33, fixed with PFA, counterstained with Hoechst 33342 to highlight nuclei, and analysed for GFP and RFP using a Zeiss LSM-700.

### Section and whole-mount *in situ* hybridisation (SISH and WISH)

SISH was performed on 10 μm paraffin sections of formaldehyde-fixed (4% PFA) organs with a digoxigenin (DIG) antisense riboprobe derived from *Fam183b* cDNA (Fantom clone; FANTOM Consortium and RIKEN Genome Exploration Research Group Phase I & II Team^45^) essentially as described^46^. WISH of mutant and wild type mouse embryos were performed in parallel under identical conditions following standard procedures. Mouse SISH and WISH results were documented with a Leica DM5000B microscope using Leica Firecam software. WISH of staged wild type and morphant *Xenopus* embryos was performed as described^47^.

### Reverse transcription (RT-) PCR from total RNA

Total RNA was isolated from dissected mouse tissues using TriReagent (Zymo Research), cDNA synthesised using SuperScriptII Reverse Transcriptase (Invitrogen), following the manufacturer’s instructions. PCR was performed using the following primer pairs:

Fam183b Ex1-F:  ATCTTGCGGGAACTCTTTC

Fam183b Ex4-B:  AGAGTGATGTCGTGGTAGAC  308 bp product (4 exon transcript)

5′HPRT:      CACAGGACTAGAACACCTGC

3′HPRT:      GCTGGTGAAAAGGACCTCT   248 bp product

### Isolation of polyA^+^ RNA and Northern Blot hybridisation

Total RNA was isolated from testis using TriReagent (Zymo Research), polyA^+^ RNA was isolated from adult wild type and *Fam183b*^*Δex1/*Δ*ex1*^ testis using magnetic Oligo dT beads (Dynal, Novagen). Northern blot hybridisation was carried out according to standard procedures. Integrity of loaded RNAs was verified by hybridisation with a β-actin probe (633 bp SalI-XbaI fragment from ZX00177K09 clone; Riken fantom II cDNA book).

### Histological methods

Immunofluorescence staining, Hematoxylin and Eosin (HE) staining and Periodic acid–Schiff (PAS) staining were performed using standard procedures.

### Sources of cDNAs and generation of expression vectors

Mouse *Fam183b* cDNA clones were obtained from the FANTOM DNA book (3 exon transcript, clone 3100002J23; Rearray ID: ZX00144L01) and from RZPD (IRALp962H0945Q, clone Image ID 6703357, Source BioScience; 4 exon transcript). Expression vectors were produced using LR clonase (Invitrogen®) according to the manufacturer’s instructions. The Fam183b ORF (4 exons), without and with STOP sequence, were PCR-amplified with primers pairs Fam183b-KpnI-ENTR1A-for (ggtaccGAATGGCCATGGCAGGACGTGTG) and Fam183boS-EcoRI-rev (gaattcTTCTGGTGATCGTCTTCCCCCAAG), or Fam183b-KpnI-ENTR1A-for (ggtaccGAATGGCCATGGCAGGACGTGTG) and Fam183bmS-EcoRI-ENTR-rev (gaattcTACTTCTGGTGATCGTCTTCCCC), cloned in pENTR^TM^1A using the KpnI/EcoRI sites following transfer into the Gateway expression vectors pCDNA6.2-N-GFP, pCDNA6.2-C-GFP (Invitogen®) and modified FLAG-vectors inwhich the GFP-ORF in pCDNA6.2-N-GFP and pCDNA6.2-C-GFP was replaced by the flag sequence. For co-IP experiments N-flag-*Fam183b* (4Exons) was cloned into pCAGGS-FLPe vector (Gene Bridges, GmbH, Heidelberg, Germany) by replacing the FLP gene of this vector by the flag-tagged *Fam183b* sequence. A mouse *Nup93* cDNA was obtained from the FANTOM DNA book (clone B230111C05; Rearray ID: PX00938F16). An expression vector for C-terminally Myc-tagged NUP93 was generated by inserting a XhoI-BlpI fragment from B230111C05 into a synthesised Nup93 cDNA fragment (accggtATGGATACTGAGGGGTTTGGTGAGCTCCTTCAGCAAGCTGAACAGCTTGCTGCTGAGACTGAAGGCATCTCTGAGCTTCCACATGTAGAACGAAATTTACAGGAGATCCAGCAAGCTGGTGAGCGCCTGCGTTCCCGTACCCTCACACGCACATCCCAGGAGACAGCAGATGTCAAGGCATCAGTTCTTCTCGGGTCAAGGGGACTTGACATATCCCATATCTCCCAGAGACTGGAGAGTCTGAGCGCAGCCACCACTTTTGAACCTCTCGAGACAAGCTGCTAAGCCCTGTTGTCCCACAGATCAGCGCCCCACAGTCTAACAAAGAAAGGCTGAAGAACATGGCCCTCTCCATTGCGGAGAGGTACAGAGCTCAGGGAATAAGTGCAAATAAGTTTGTGGACTCTACATTCTATCTCCTCTTGGACCTGATCACCTTTTTTGACGAGTATCACAGTGGTCATATTGACAGAGCCTTTGATATTATTGACCGCTTGAAGCTGGTGCCTCTGAATCAGGAGAGTGTGGAAGAAAGGGTGGCTGCCTTCAGAAACTTCAGTGATGAAATCAGACACAACCTCTCAGAAGTTCTTCTCGCCACCATGAACATCCTGTTCACACAGTTTAAGAGGCTCAAAGGAACAAGTCCATCTTCAGCAACCAGGCCCCAGCGAGTCATTGAAGACCGTGACTCTCAACTCCGAAGTCAAGCCAGAGCCCTGATTACCTTTGCTGGGATGATACCGTACCGGACGTCGGGGGACACTAATGCCAGGCTGGTGCAGATGGAGGTCCTCATGAATGCATCAGAGCAGAAGCTGATCTCAGAGGAGGACCTGCAgcggccgc) and cloning the Nup93-Myc coding sequence as AgeI-NotI fragment into pIRESpuro3 (Clontech #631619). For the Y2H screen, the ORF of *Fam183b* was flanked by EcoRI and SalI sites by PCR using primers Fam183b-EcoRI-Y2H#2431-for (gaattcATGGCCATGGCAGGACGTGTGGGGC) and Fam183b-SalI-Y2H#2431-rev (gtcgacTCACTTCTGGTGATCGTCTTCCCCC) and cloned into the bait-vector pGBT9 DNA-BD (Clontech #K1605-A). Expression vectors for Strep/Flag-tagged FAM183b were generated by flanking the Fam183b ORF (4 Exon transcript) with KpnI and EcoRI, or NheI and XhoI sites by PCR using primers C-SF-Fam183b-KpnI-for (ggtaccCATGGCCATGGCAGGACGTGTGGGGC) and C-SF-Fam183b-EcoRI-rev (gaattcTCGAGCTTCTGGTGATCGTCTTCCCCC) and N-SF-Fam183b-NheI-for (gctagcATGGCCATGGCAGGACGTGTGGGGC) and N-SF-Fam183b-XhoI-rev (ctcgagCTACTTCTGGTGATCGTCTTCCCCC), respectively, and cloned into N-SF-TAP pcDNA3 and C-SF-TAP pcDNA3^31^. All constructs were sequence-verified.

### Yeast-two-Hybrid Screening

The Yeast-two-Hybrid screen was performed by the DKFZ Protein-Protein Interaction Screening Facility as described^48^. As bait, the ORF of *Fam183b* (3 exon transcript) fused to the GAL4 DNA binding domain was used to screen a mouse testis prey library (Clontech) in histidine/adenine free medium in the presence of 2 mM amino-triazole.

### Affinity purification and mass spectrometry

N- and C-terminally tandem StrepII-Flag-tagged FAM183b (4 exon variant) was transiently expressed in HEK293T cells, FAM183b complexes were isolated^31^ and analysed by LC-MS/MS as described^49^. Selected candidate interaction partners identified by MS were further analysed by Co-IP.

### Co-IP of overexpressed proteins from CHO cells

Expression plasmids for Flag-tagged FAM183b (in pCAG-IRESpuro) and Myc-tagged NUP93 (in pIRESpuro3) were co-transfected into CHO cells (ATCC® CCL-61™), using PerFectin transfection reagent (Genlantis) according to the manufacturer’s instructions. Cells were harvested 16 hrs after transfection, washed on ice with cold PBS, covered by lysis buffer (50 mM Tris-Hcl pH7,5, 150 mM NaCl, 1 mM EDTA, 1% NP-40, 1% Triton X-100, 1% BSA, 1x Complete EASY proteinase inhibitor (Roche, #04693159001, 500 μl per 10 cm dish), and incubated for 5 min on ice. Cells were scraped off the dish and genomic DNA was sheared by pulling through a cannula (BRAUN-9186166), incubated for 20 min on ice, and centrifuged 15 min at 4 °C and 3000 rpm. 25 μl Protein G-Sepharose beads (Amersham, #17-0618-01) was added to the supernatant and incubated for 1 h at 4 °C with continuous inversion for preclearing. The preclearing step was repeated once and beads were pelleted by centrifugation (2 min at 3000 rpm). 50 μl supernatant was removed as input control, the remainder distributed equally to three vials and filled up to 1 ml with 20 mM NaPi pH7,0 (with proteinase inhibitor). Vials were incubated with either 8 μl mouse anti-Myc 9E10 (Sigma, M5546, 6,1 mg/ml) or mouse anti-Flag M2 (Sigma, F3165, 4,2 mg/ml) or without antibody 1,5–3 h on a turning wheel at 4 °C. 25 μl Protein G-beads (GE healthcare, 17-0618-01) per IP were preincubated 1,5–3 h in 1 ml 20 mM NaPi pH7,0 with 1% BSA (with proteinase inhibitor) on a turning wheel at 4 °C, harvested by centrifugation, added to each IP sample and incubated on a turning wheel at 4 °C overnight. IPs were washed three times for 20 min on a turning wheel with wash buffer (50 mM Tris-HCl pH8.5, 500 mM NaCl, 5 mM EDTA, 0,05% NP-40, 0,1% BSA, 1x proteinase inhibitor), the supernatant was completely removed from beads, 40 μl 2x sample buffer added (125 mM Tris-HCl pH 6,8, 20% glycerol, 4% SDS, 0,04% Bromphenol Blue, 2% β-mercaptoethanol, 200 mM DTT, 10 mM Iodoacetamid), heated 5 min to 95 °C and 20 μl loaded on SDS-PAGE. Western blots were performed according to standard procedures. Membrane was cut horizontally at the 50 kDa marker band. Protein on the upper part was detected with anti-Myc-POD (Sigma, A5598) on the lower part with anti-Flag-POD (Sigma, A8592) 1:10000 in 5% milk/0,1% Tween20 in PBS. Pictures were obtained in a Fuji LAS4000 chemiluminescence reader.

### High-speed video microscopy of epidermal cilia

Wild type or morphant specimens were analysed for epidermal ciliary beat patterns at stage 33. Embryos were mounted on a slide containing a rectangular chamber constructed from duct tape. Ciliary beating was recorded using a high-speed Hamamatsu video camera Orca flash 4.0 at 600 frames per second.

### Measurements and statistics

Measurements were performed in ImageJ (https://imagej.nih.gov/ij/). p-values for ordinal data were calculated using the two-sided Fisher’s exact test (https://www.graphpad.com/quickcalcs/contingency1/). p-values for metric data were determined using the Mann-Whitney U test in R (https://www.r-project.org/). Significance was scored as follows: p ≥ 0.05: not significant; *p < 0.05: **p < 0.01: ***p < 0.001.

### Multiple sequence alignments and phylogenetic analysis

Sequences were aligned using ClustalW (v1.83; multiple sequence alignment; Pairwise Alignment Mode: Slow; Pairwise Alignment Parameters: Open Gap Penalty = 10.0, Extend Gap Penalty = 0.1, Similarity Matrix: gonnet; Multiple Alignment Parameters: Open Gap Penalty = 10.0, Extend Gap Penalty = 0.2, Delay Divergent = 30%, Gap Distance = 4; Similarity Matrix: gonnet). The phylogenetic tree was build by Neighbor joining, Best Tree Mode (systematic tie breaking). Distances were estimated and gaps distributed proportionally. The tree was rooted to human FAM183A.

## Electronic supplementary material

Supplementary Information

Movie S1 wild type

Movie S2 SBMO

Movie S3 TBMO

## Data Availability

The data generated or analysed during this study are included in this published article (and its Supplementary Information files), the full mass spectrometry proteomics data have been deposited to the ProteomeXchange Consortium via the PRIDE^50^ partner repository with the dataset identifier PXD009409 (Username: reviewer22871@ebi.ac.uk Password: UVNiVt93).
