# Supplementary material for: The evolutionary conserved FOXJ1 target gene Fam183b is essential for motile cilia in Xenopus but dispensable for ciliary function in mice
Source: Sci Rep. 2018 Oct 2;8:14678. doi: 10.1038/s41598-018-33045-2 (PMC6168554; doi:10.1038/s41598-018-33045-2)
Supplement: Supplementary file 1 — Supplementary Information [file 41598_2018_33045_MOESM1_ESM.pdf]

SUPPLEMENTARY INFORMATION

The evolutionary conserved FOXJ1 target gene *Fam183b* is essential for motile cilia in *Xenopus* but dispensable for their function in mice

Anja Beckers, Tim Ott, Karin Schuster-Gossler, Karsten Boldt, Leonie Alten, Marius Ueffing, Martin Blum and Achim Gossler

This file contains Supplementary Figures S1-S5 and Supplementary Tables S1-S3

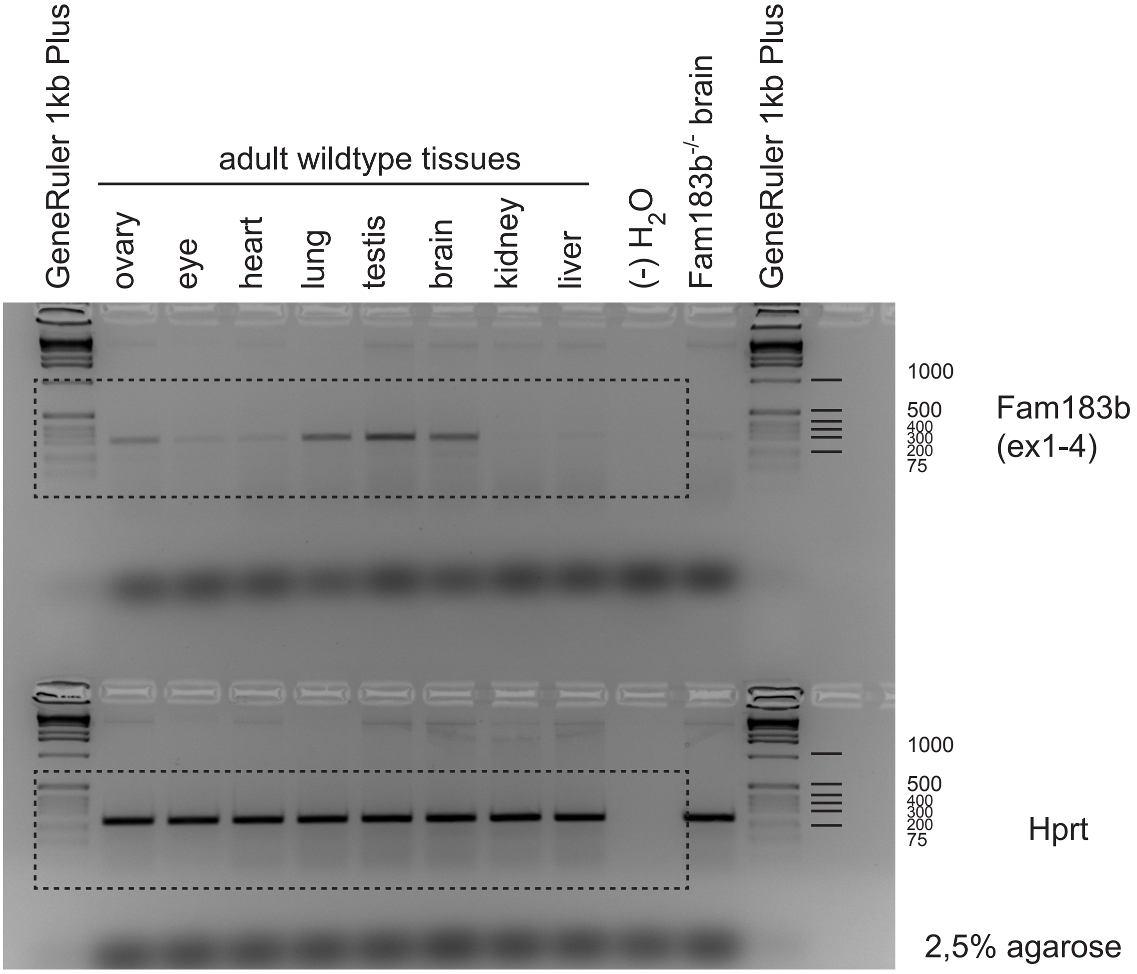

Supplementary Figure S1. Photo of the full size gel of the cropped panels (stippled rectangles) shown in Fig 1C.

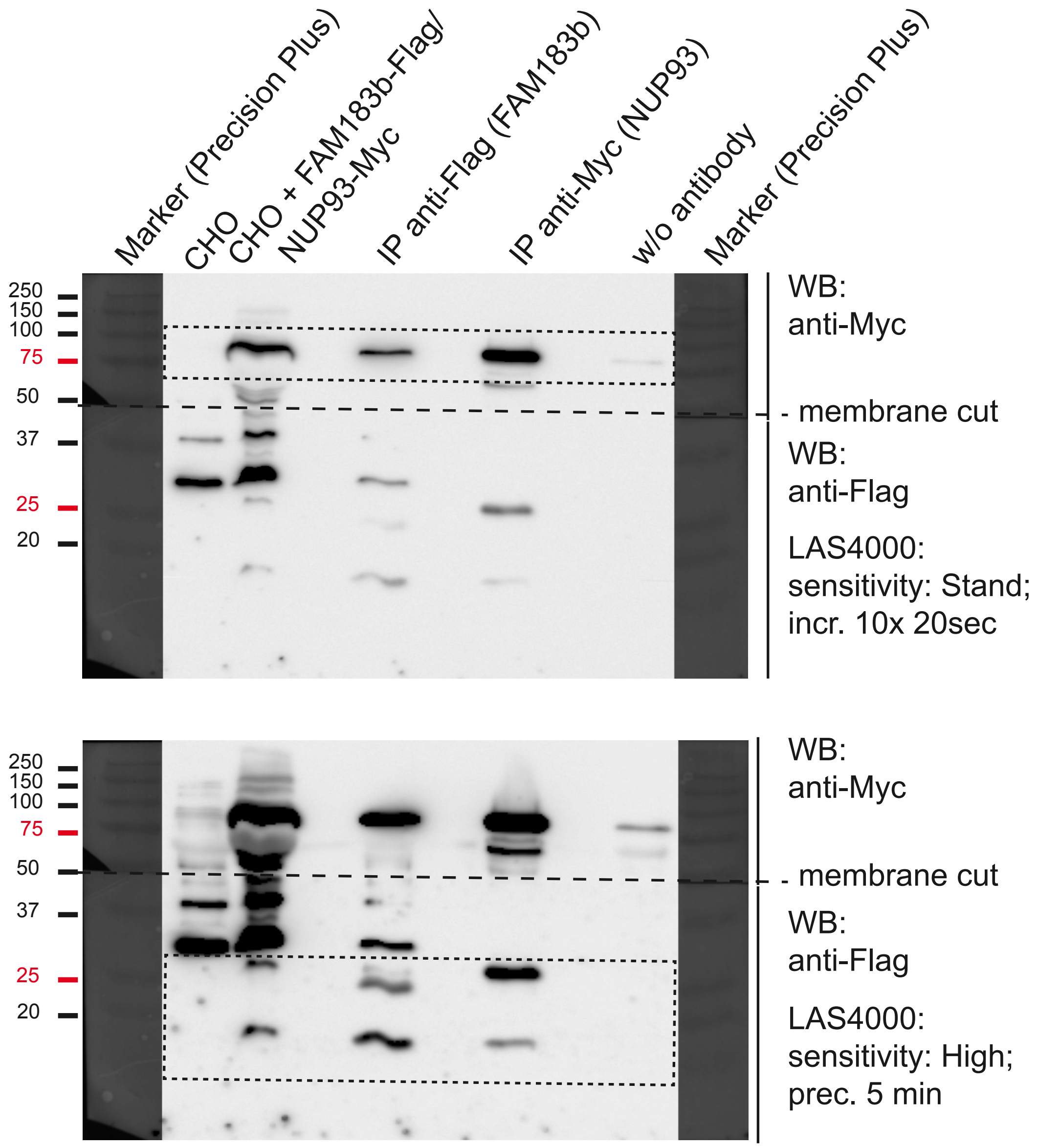

Supplementary Figure S2. Different exposures of the full size Western blots of the cropped panels (stippled rectangles) shown in Fig 2C. The membrane was cut as indicated at the stippled line, the upper part probed with anti-Myc, the lower part with anti-Flag antibodies.

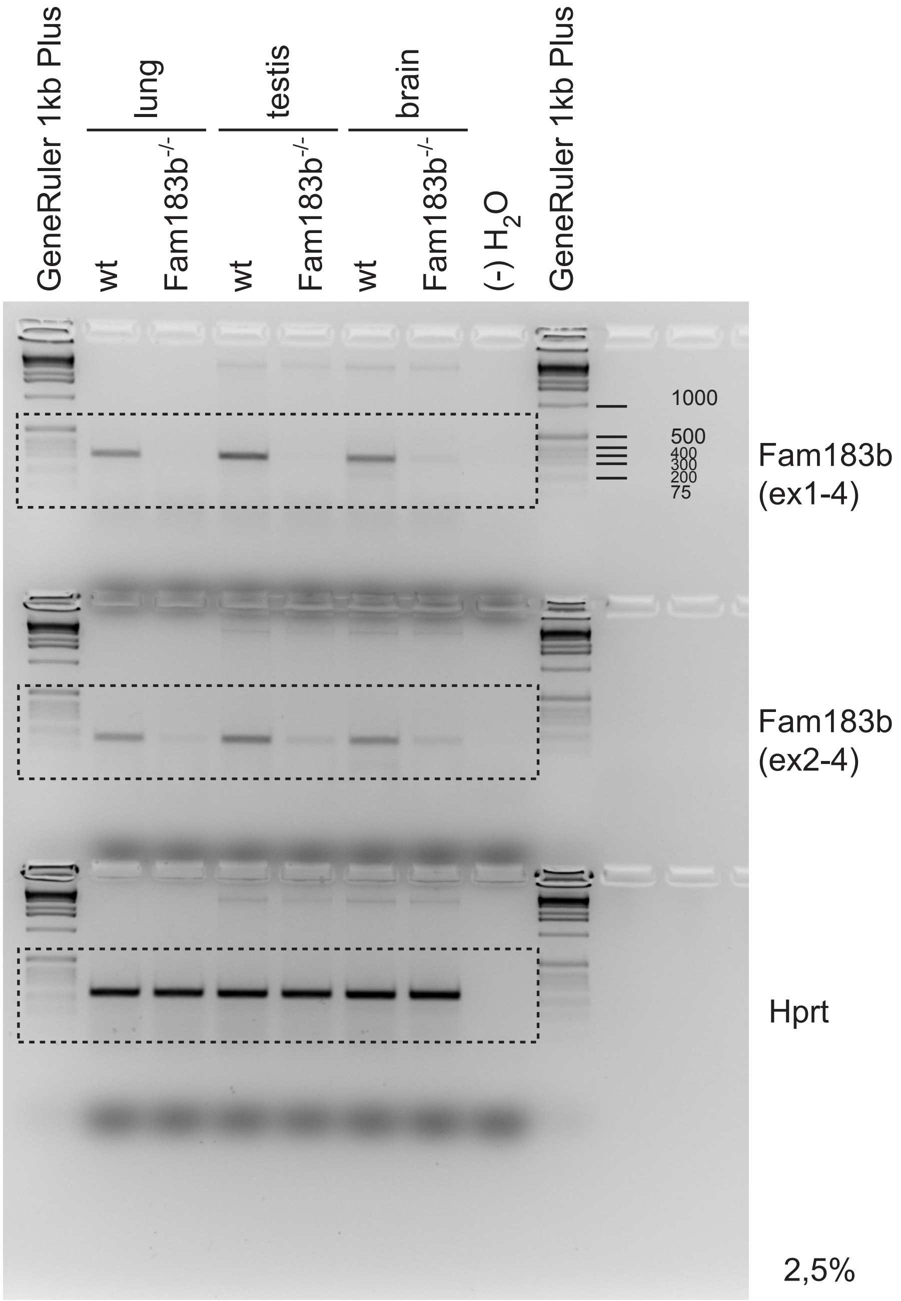

Supplementary Figure S3. Full size gel of the cropped panels (stippled rectangles) shown in Fig 3B.

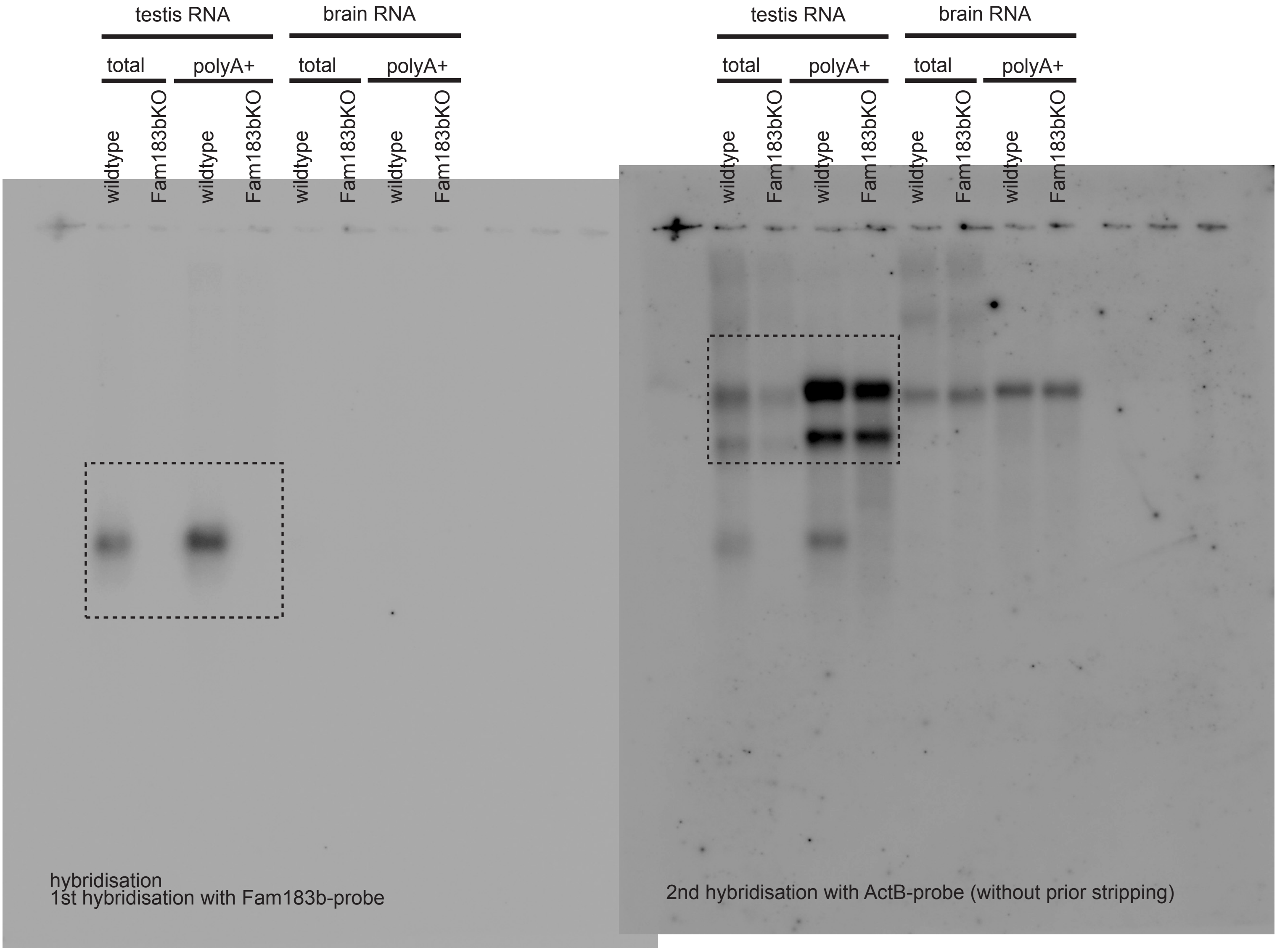

Supplementary Figure S4. Full size Northern blots of the cropped panels (stippled rectangles) shown in Fig 4C.

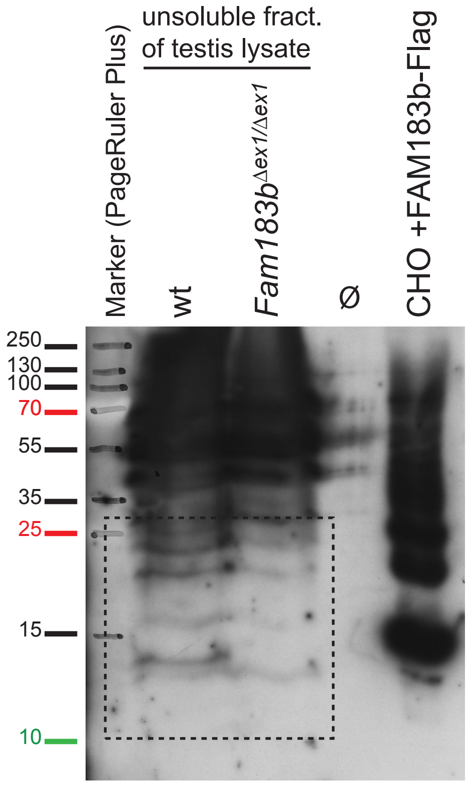

Supplementary Figure S5. Full size Western blot of the cropped panel (stippled rectangle) shown in Fig 4E.

		** Identity Scores (%) **										
		human	mouse	chick	Xenopus	zebrafish	ciona	branchiostoma	nemostella	hydra	Sea urchin	Chlamydomonas
Human_NP_001098752_1	** Similarity Scores (%) **	100.0	76.6	51.1	49.3	43.4	41.9	37.0	38.0	35.3	34.5	18.1
Mouse_NP_083559_1		86.1	100.0	51.8	48.6	42.0	43.5	43.5	39.1	37.0	38.8	15.2
Gallus_XP_422395_1		67.4	66.4	100.0	55.1	47.1	40.4	41.3	35.8	37.5	38.8	16.1
Xen_trop_XP_002931631_1		62.5	62.3	65.4	100.0	57.4	49.3	50.7	46.0	44.1	47.5	17.4
Zebrafish_XP_001332180_3		59.6	58.7	61.8	76.5	100.0	61.5	58.7	50.4	41.2	51.8	16.9
Ciona_XP_002127995_1		54.4	55.1	55.1	68.4	74.8	100.0	76.1	66.4	52.2	71.9	18.2
Branchiostoma_XP_002603871_1		52.9	56.5	55.1	66.7	73.2	88.4	100.0	64.5	52.2	71.9	16.6
Nemostella_XP_001637177_1		54.0	52.9	53.3	65.7	66.4	82.5	80.4	100.0	56.2	64.0	16.7
Hydra_XP_002162150_1		54.4	52.2	52.9	60.3	62.5	68.4	69.6	73.0	100.0	51.1	19.5
Sea urchin_XP_003728346		52.5	55.4	55.4	66.2	67.6	83.5	87.8	81.3	67.6	100.0	16.4
Chlamydomonas_ACJ06133		30.9	28.5	32.9	34.9	33.1	36.5	34.4	32.0	35.6	32.9	100.0

Supplementary Table S1. Similarity matrix of FAM183b amino acid sequences from different species based on the alignment shown below. Sequences were aligned using ClustalW (v1.83; multiple sequence alignment; Pairwise Alignment Mode: Slow; Pairwise Alignment Parameters: Open Gap Penalty = 10.0, Extend Gap Penalty = 0.1, Similarity Matrix: gonnet; Multiple Alignment Parameters: Open Gap Penalty = 10.0, Extend Gap Penalty = 0.2, Delay Divergent = 30%, Gap Distance = 4; Similarity Matrix: gonnet).

Formatted Alignments

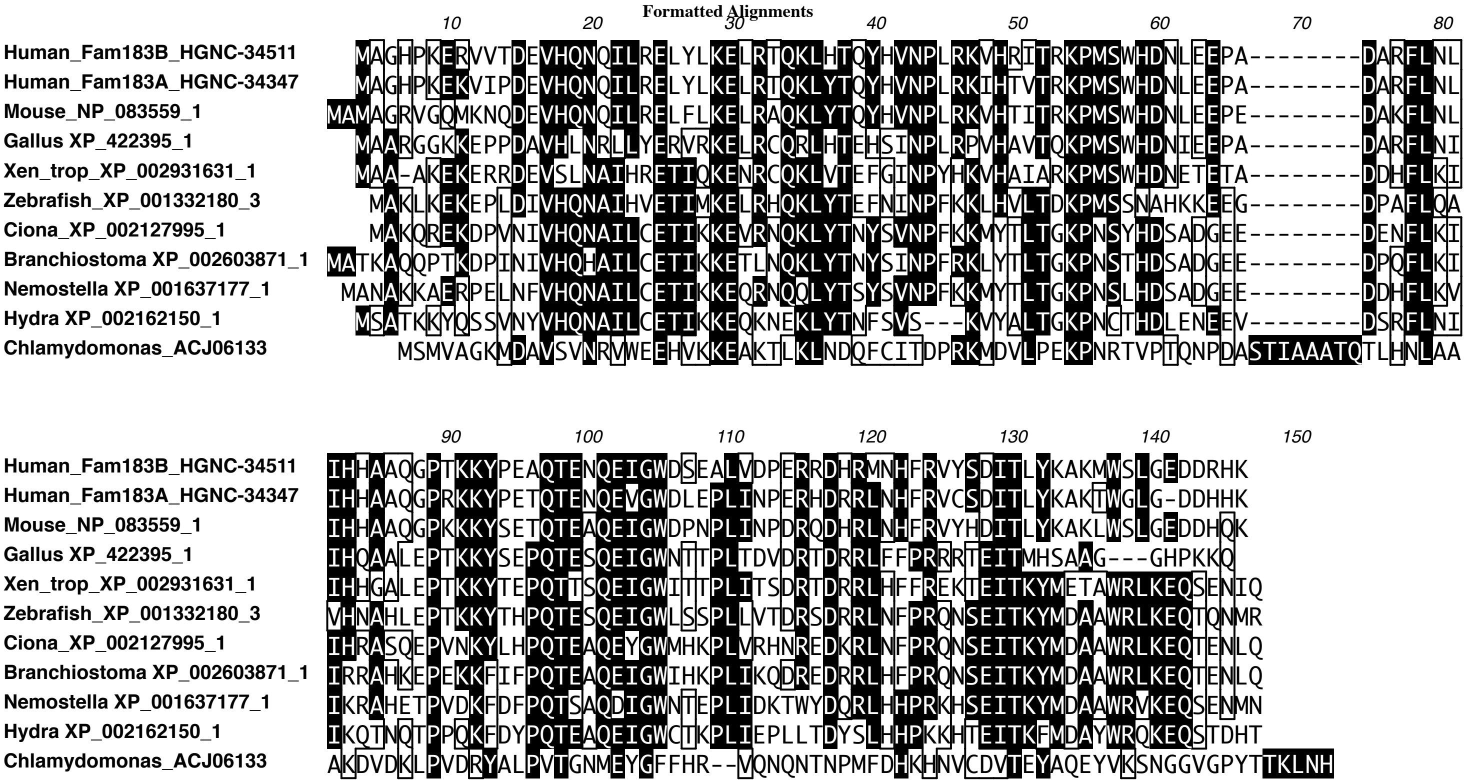

Prey Protein		Accession #	# of isolates	found in # of screens
NUP93	Nucleoporin 93	Q8N1F7	2	2
TMEM269	transmembrane protein 269	A0A1B0GVZ9	2	2
ANKRD36	ankyrin repeat domain 36	A6QL64	2	1

Supplementary Table S2. Prey proteins identified in a Y2H screen using FAM183b as a bait.

			Number of unique peptides					
			FAM183b NTAP			FAM183b CTAP		
			Experiment #			Experiment #		
**Protein name**	**Accession Number**	**Molecular Weight**	**1**	**2**	**3**	**1**	**2**	**3**
Protein FAM183B	F183B_MOUSE	16 kDa	11	12	10	12	12	12
Probable serine carboxypeptidase CPVL	CPVL_HUMAN	54 kDa	40	44	64	28	27	31
Heat shock 70 kDa protein 4L	HS74L_HUMAN	95 kDa	14	15	16	21	19	21
Exportin-T	XPOT_HUMAN	110 kDa	5	5	2	5	7	4
Calumenin	CALU_HUMAN	37 kDa	5	6	5	7		3
Fanconi anemia group I protein	FANCI_HUMAN	149 kDa	4	8	4	4	5	3
Serine/threonine-protein phosphatase PGAM5, mitochondrial	PGAM5_HUMAN	32 kDa	3	12	14		3	2
Excitatory amino acid transporter 1	EAA1_HUMAN	60 kDa	3	4	5			
Inter-alpha-trypsin inhibitor heavy chain H2	ITIH2_HUMAN	106 kDa	2	3	3	5	12	9
Filaggrin-2	FILA2_HUMAN	248 kDa	2	6	3	2	4	3
Alpha-galactosidase A	AGAL_HUMAN	49 kDa	2		3			
Proteasome subunit beta type-6	PSB6_HUMAN	25 kDa	2	2				
Filaggrin	FILA_HUMAN	435 kDa	2	2			2	
ATPase family AAA domain-containing protein 3B	ATD3B_HUMAN	73 kDa	2	5			3	2
Calcium-binding mitochondrial carrier protein Aralar2	CMC2_HUMAN	74 kDa		15	3	4	21	6
Heat shock 70 kDa protein 1-like	HS71L_HUMAN	70 kDa		3	3	3	4	4
Mitochondrial glutamate carrier 1	GHC1_HUMAN	34 kDa		4	4	5	5	4
Glutamate dehydrogenase 1, mitochondrial	DHE3_HUMAN	61 kDa			3	5		6
Nuclear pore complex protein Nup93	NUP93_HUMAN	93 kDa		3			6	3
ATP synthase subunit gamma, mitochondrial	ATPG_HUMAN	33 kDa		2			5	2
RING finger protein 219	RN219_HUMAN	81 kDa		3			3	3
Sideroflexin-4	SFXN4_HUMAN	38 kDa			2	2	3	3
Heat shock-related 70 kDa protein 2	HSP72_HUMAN	70 kDa			2		2	2
40S ribosomal protein S3	RS3_HUMAN	27 kDa		2		2		3
Tetratricopeptide repeat protein 27	TTC27_HUMAN	97 kDa		2		2	2	
Very-long-chain 3-oxoacyl-CoA reductase	DHB12_HUMAN	34 kDa		2		2	2	
Insulin-degrading enzyme	IDE_HUMAN	118 kDa				35	68	62
E3 ubiquitin-protein ligase UBR2	UBR2_HUMAN	201 kDa				14	17	7
Serine/threonine-protein phosphatase 2A 55 kDa regulatory subunit B delta isoform	2ABD_HUMAN	52 kDa					4	4
DNA damage-binding protein 1	DDB1_HUMAN	127 kDa				3	2	
Serine/threonine-protein phosphatase 2A 65 kDa regulatory subunit A beta isoform	2AAB_HUMAN	66 kDa				3	2	
Fatty acyl-CoA reductase 1	FACR1_HUMAN	59 kDa					2	2

Supplementary Table S3. Proteins identified in mass spectrometry analyses of purified complexes of N- and C-terminally TAP-tagged FAM183b. Shown are hits that were obtained at least in two independent experiments and absent from controls. The full mass spectrometry proteomics data have been deposited to the ProteomeXchange Consortium via the PRIDE partner repository with the dataset identifier PXD009409

Movie Legends

Movie S1. Wild type ciliary beating.

Time-lapse video of ciliary beating of multi-ciliated epidermal cells recorded at stage 33 from wild type specimens. Movie was recorded at 600 frames per second and plays at 0.05 x real time.

Movie S2. Impaired ciliary beating in *Fam183a*-morphant injected with translation-blocking MO.

Time-lapse video of ciliary beating of multi-ciliated epidermal cells recorded at stage 33 from *Fam183a*-TBMO-injected specimen. Movie was recorded at 600 frames per second and plays at 0.05x real time.

Movie S3. Impaired ciliary beating in *Fam183a* -morphant injected with splice-blocking MO.

Time-lapse video of ciliary beating of multi-ciliated epidermal cells recorded at stage 33 from *Fam183a*-SBMO-injected specimen. Movie was recorded at 600 frames per second and plays at 0.05x real time.
